# Efficacy and Safety of Kleeb Bua Daeng Formula in Mild Cognitive Impairment Patients: A Phase I Randomized, Double-Blind, Placebo-Controlled Trial

**DOI:** 10.1155/2022/1148112

**Published:** 2022-03-25

**Authors:** Natdanai Musigavong, Chantana Boonyarat, Yaowared Chulikhit, Orawan Monthakantirat, Makorn Limudomporn, Supaporn Pitiporn, Pakakrong Kwankhao, Supawadee Daodee

**Affiliations:** ^1^Faculty of Pharmaceutical Sciences, Khon Kaen University, Khon Kaen 40002, Thailand; ^2^Department of Neurology, Chao Phya Abhaibhubejhr Hospital, Prachinburi 25000, Thailand; ^3^Center of Evidence-Based for Thai Traditional and Herbal Medicine, Chao Phya Abhaibhubejhr Hospital, Prachinburi 25000, Thailand

## Abstract

Individuals with mild cognitive impairment (MCI) were at increased risk of conversion to dementia. The Kleeb Bua Daeng (KBD) formula could be the alternative treatment option for MCI through multitarget activities. Lacking of clinical trial information brought about the study in our research. Forty patients with MCI were randomly assigned to receive the KBD capsule or placebo at a dose of 1,000 mg twice a day for three months. Their cognitive functions were monitored by the Montreal Cognitive Assessment (MoCA) and blood chemistry assessment every one month. We found that the KBD-treated group had no significant differences in the MoCA test compared to placebo. Moreover, there was no alteration in biochemical parameters of the liver and renal function was observed which could confirm the safety of this KBD formula.

## 1. Introduction

Mild cognitive impairment (MCI) represents a clinical construct that identifies an intermediate state of cognitive function between healthy aging and memory and cognitive deficits associated with frank dementia. Reviews of several studies have indicated that the individuals with MCI are at an increased risk for developing any form of dementia ranging from 12% to 20% per year [1]. The risk of MCI is higher with lower education, increasing age, and male gender [2]. Presently, there is no evidence for the use of medications as disease modifiers. However, the absence of demonstrated efficacy in MCI should not be a barrier to these interventions [3]. The pharmacological treatment was only approved for mild to moderate Alzheimer's disease (AD). The acetylcholinesterase inhibitors were not effective for AD and have various side effects such as nausea, bradycardia, and fatigue [4]. The exercise has beneficial effects for a large number of conditions affecting the elderly and may be incorporated into the treatment of MCI. The patients should engage in at least three half-hour sessions of aerobic exercise weekly.

The pathological profile of MCI is heterogeneous, and many lesions seem to be on a continuum between dementia and cognitively intact individuals [5]. Brain amyloid-beta (A*β*) plaques are a hallmark lesion of people with a clinical diagnosis of MCI. The distribution of A*β* deposits changes with time and reflects the spread of A*β* deposition in the diseased brain [6]. Senile plaques first appear as diffuse and “fleecy” plaques throughout the neocortex and extend hierarchically into other brain regions. Additionally, the oxidative stress may stimulate alteration in glutamate signaling, cause calcium ion influx to cell, and then urge many enzymes such as phospholipase, protease, nitric oxide synthase, protein kinases, and xanthine oxidases that give free radical and result in neurodegeneration [7]. Therefore, MCI patients show a more significant symptom of memory deficit when compared with normal subjects with little or absent involvement in typical activities of daily living.

Kleeb Bua Daeng formula (KBD) is composed of three dried medicinal herbs in 1 : 1 : 1 ratio: petal of *Nelumbo nucifera* Gaertn., leaf and stem of *Centella asiatica* (L.) Urb., and fruit of *Piper nigrum* L.. This formula has shown health benefits on patients according to Thai traditional medicine practitioner's observation. *N. nucifera* has been investigated on activities related to neurodegeneration conditions such as AD. From the *in vitro* study, *N. nucifera* exhibited the potential multipoint of activities, including inhibition of enzymes implicated, both AChE and BChE, *β*-site APP cleaving enzyme 1 (BACE1) inhibition, and antioxidant [8], whereas from the *in vivo* study antiamnesia result, the *N. nucifera* may reverse scopolamine-induced cognitive dysfunction by increasing the number of ChAT-positive neurons' expression and AChE-inhibitory activities [9]. Moreover, *C. asiatica* and *P. nigrum* have been confirmed to possess good antioxidant activity, inhibit the activity of the enzyme cholinesterase, and against beta-amyloid aggregation, which is a significant pathogen of AD. In previous studies, the original KBD formula was scientifically proved by *in vitro* and *in vivo* models to benefit neuroprotective effect and safety [10, 11].

This clinical trial study was performed as a phase 1 trial composed of people with the MCI symptom. This clinical trial evaluated its potential for improving the cognitive function and safety of the KBD formula in a small group of people. MCI patients were selected due to the significant risk of progression to Alzheimer's disease, which the early treatment could reduce progression to more severe cognitive impairment.

## 2. Materials and Methods

### 2.1. Study Protocol

This study was performed according to the guidelines of Helsinki Declaration and approved by the Center for Ethics in Human Research, Khon Kaen University (HE631014). Participants were fully informed of the intervention protocol, and they signed the written informed consent forms. The trial was registered at Thai Clinical Trials Registry (https://www.thaiclinicaltrials.org; registration number, TCTR20220209007; registration date, 9 February 2022).

### 2.2. Randomization and Blinding

In this randomized, double-blinded, placebo-controlled clinical trial, MCI patients in Chao Phya Abhaibhubejhr Hospital, Prachinburi Province, Thailand, were enrolled. For group allocation, simple randomization procedure was used. The random sequence was generated by someone who was not involved in the intervention. The allocation was concealed till the end of the study. Each drug (KBD formula or placebo) and its placebo had identical shape, color, size, and packaging and also coded with sequential numbers by someone who was not involved in the intervention. The interpretation of codes was concealed to the researchers till the end of the analysis. All investigators, participants, and the laboratory technicians were blinded to the random assignments and content of the intervention.

### 2.3. Medicine Preparation

KBD formula containing 166.67 mg of *N. nucifera* petal, *C. asiatica* aerial part, and *P. nigrum* fruits at 1 : 1 : 1 ratio was assembled from Chao Phya Abhaibhubejhr hospital foundation (Lot number HB90120001), Thailand. The same place made the placebos. Placebos were the same as the original drug in shape, color, size, and packaging. The HPLC analysis of the KBD extract had been reported in a previous study [11].

### 2.4. Participants and Study Design

In this research, MCI patients in Chao Phya Abhaibhubejhr Hospital, Prachinburi of Thailand, were enrolled. The inclusion criteria were as follows: provision of signed informed consent before any study-specific procedure, MoCA (Montreal Cognitive Assessment)-T score between 19 and 24, and 40–70 years of age. The exclusion criteria were as follows: GFR by Cockcroft formulation ≤40 ml/min, serum alanine aminotransferase (ALT) and aspartate aminotransferase (AST) higher than 2.5 of standard level, patients who receive warfarin, pregnancy or breastfeeding, need for or an anticipated need for oral with any of the following: (a) cholinesterase inhibitors, (b) N-methyl D-aspartate antagonist (NMDA), and (c) cholinesterase inhibitors plus NMDA, and participation in another clinical study with an investigational product at any time during the 30 days before randomization. The selected participants (based on inclusion criteria) were randomly divided into two equal groups: the KBD group, which received two capsules twice a day; the placebo group, which received two capsules twice a day. Patients were visited every one month. To determine the medication adherence of KBD formula and placebo, the research team asked the participants to keep an empty bottle of consumed medicines and deliver them on the visits.

The sample size was calculated as the estimated sample size for repeated-measures ANOVA by STATA software. Since 20% of subjects are expected to be lost to follow-up, the total sample size was adjusted to 38.4. Thus, our study used a total sample size of forty volunteers. To our knowledge, this study was the first clinical trial of KBD for mild cognitive impairment, so our study has chosen the study [12] as a reference for sample size calculation. The previous study was aimed to investigate the efficacy of the herbal product for vascular dementia. The herbal product showed similar pharmacologic properties. In addition, the pathogenesis of vascular dementia was similar to mild cognitive impairment via the abnormal cholinergic system and brain biomarkers.

### 2.5. Statistical Analyses

Quantitative data were presented as mean ± SD, and qualitative data were presented as number and percentage. Normality of studied variables was assessed by the Kolmogorov–Smirnov test and Q-Q plot. The significant differences were compared between control and treatment. The difference among various groups was measured by ANOVA (post hoc test), independent *t*-test, and mixed linear regression model. Fisher's exact test was performed for categorical data. The statistical difference will be regarded at *p* < 0.05.

## 3. Results

**Figure 1:**
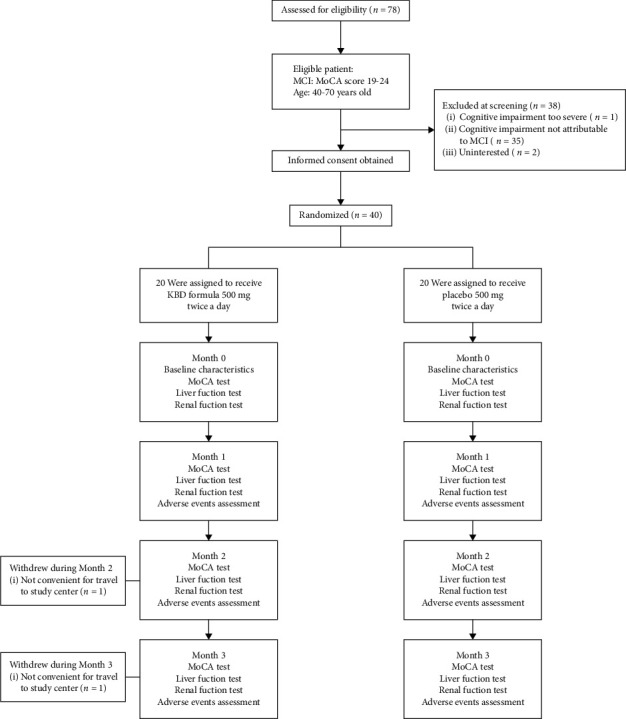
CONSORT flowchart of the KBD clinical trial.

A total of 78 individuals were assessed for study eligibility. The flow of participants from screening to enrollment is shown in Figure 1. Participants (*n* = 40) were randomized to either the KBD (*n* = 20) or placebo (*n* = 20) groups. All participants were diagnosed with mild cognitive impairment. A total of 38 participants (95%: KBD *n* = 18, placebo *n* = 20) completed the study (Figure 1).

Demographic and baseline characteristics are given in Tables 1 and 2. There are no significant differences in the two groups.

The KBD group averaged 93.09% (SD 3.33%) of the medication adherence as calculated by pill count in each visit, and the placebo group completed 95.22% (SD 3.01%) of the medication adherence.

**Figure 2:**
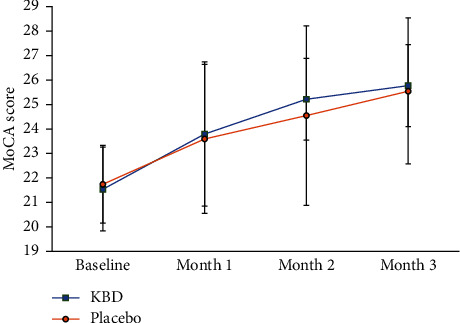
Estimated primary outcome effects of difference between KBD and placebo.

The KBD group was associated with a significant gain in MoCA score at month 3 when compared to baseline. Although the result of MoCA score was higher in every visit in the KBD group, there was no difference in primary outcome measures when compared to the placebo group at month 3 (Figure 2) (Tables 3 and 4).

For secondary outcome, we assessed the safety profile of KBD by evaluating the liver function and kidney function. The average dose per day of KBD was 3.72 ± 0.13 capsules. The liver safety data were crucial for new therapeutic agents in clinical trial. All participants in both groups have normal values of liver enzymes at baseline. There was no significant difference among the groups at baseline. All serial liver tests presented the normal biochemical indicator in both groups. There was no difference between groups at each time point (Table 5). At the end of the study, the KBD and placebo showed no significant difference in AST and ALT levels.

Drug-induced kidney injury was investigated for human safety. In this study, both groups were observed with normal renal function at the beginning. No difference was observed in serum creatinine at different time points between two groups (Table 6). On KBD, the participants reported a normal serum creatinine level at month 3.

Another standard kidney biomarker was eGFR which can indicate a clinically significant loss of kidney function. A decrease in eGFR of more than 5 mL/min/1.73 m^2^ could have a clinical impact on participants. At baseline, both groups have normal renal function. At different time points, there was no significant difference in eGFR level between groups (Table 7). By determining the eGFR level, it is found that the KBD did not have a significant effect on kidney function.

There were five adverse events possibly related to the intervention. Common mild adverse events include feeling hot in the throat (*n* = 3), dizziness (*n* = 1), and drowsiness (*n* = 1) in the KBD group. No severe adverse event was present in this study (Table 8).

## 4. Discussion

Presently, the agents with a single mode of action may have limited impact on AD with mixed pathologies. Because of the benefits of the therapeutic effect of KBD on cognitive function both *in vitro* and *in vivo*, the clinical trial was conducted in order to explore its safety and effectiveness. This clinical trial study was performed as a phase 1 trial composed of people with the disease. MCI patients were selected due to the significant risk of progression to Alzheimer's disease, which the early treatment could possibly reduce progression to more severe cognitive impairment. The pathological profile of mild cognitive impairment was heterogeneous, and many lesions seem to be on a continuum between dementia and cognitively intact individuals [5]. Brain amyloid beta plaques were a hallmark lesion of people with a clinical diagnosis of mild cognitive impairment. The distribution of amyloid beta deposits changes with time and reflects the spread of amyloid beta deposition in the diseased brain [6]. In those with MCI versus age-matched participants at 2–5 years after, the relative risk of dementia was 3.3 (95% CI: 2.5–4.5, I2 = 4.9) and the relative risk of the diagnosis of AD was 3.0 (95% CI: 2.1–4.8, I2 = 17.3) [2]. In the current study, all mild cognitive impairment participants were diagnosed with MoCA score (MoCA-Thai) and clinical symptoms by a neurologist. There was no significant learning effect at one month for the MoCA retest. This cognitive assessment tool was proved to investigate mild cognitive impairment in the Thai population [13]. There were three possible outcomes after patients were diagnosed with mild cognitive impairment, including reverse to normal, stable, and progression to dementia [14]. However, mild cognitive impairment patients have presented higher mortality than normal subjects [15]. The acetylcholinesterase inhibitors donepezil and galantamine were introduced to use in mild cognitive impairment, but both have a more adverse effect than benefits [16]. In a previous study, the research presented the potential mechanisms and efficacy for KBD to prevent or treat AD, which could be used in patients with memory deficits [10]. Furthermore, the oral administration of KBD showed significantly improved cognitive function in chronic mild stress mice [11].

At present, there is no approval for over-the-counter supplements or drugs to prevent or treat mild cognitive impairment [17]. This randomized, controlled trial provides the first scientific evidence that 3 months of KBD benefit from improved MoCA scores in mild cognitive impairment but not significantly different from placebo.

The pharmacologic effects are thought to be utilized by its constituents. The KBD was indicated as a multimode of action against AD via high phenolic and flavonoid content [10]. Also, HPLC analysis of the KBD extract presented the madecassoside, piperine, luteolin-7-O-glucoside, asiaticoside, rutin, quercetin, kaempferol-3-glucoside, ferulic acid, and kaempferol as major constituents [11]. Previous studies demonstrated that KBD has many pharmacological mechanisms including antioxidant activities, inhibition of *β*-amyloid aggregation, neuroprotection, antiapoptosis, and cholinesterase inhibition activity which helped improve memory deficit in mice [10]. Therefore, the improvement in cognitive impairment might partly occur via a mechanism from their study. Furthermore, this research might suggest that KBD use in mild cognitive impairment could affect pathological change but not symptomatic change.

These clinical trials in adults with mild cognitive impairment found no difference in cognitive benefit between KBD and placebo groups after a follow-up of 3 months which resembles the previous largest trial of *Ginkgo biloba* called The Ginkgo Evaluation of Memory (GEM study), which found that *Ginkgo biloba* does not provide cognitive benefit to the patient with mild cognitive impairment [18]. Previous studies found that *Ginkgo biloba* plant extract named EGb 761^®^ has a moderate effect on the cognitive function of AD patients. The EGb 761 showed a similar mechanism of action as KBD [19]. However, the mild cognitive impairment treatment recommendations reported internationally are inconsistent, but a clinical expert in Asia recommended EGb 761^®^ for multidomain intervention for mild cognitive impairment [20]. *Bacopa monniera* has been widely used for cognitive improvement as an alternative choice. The *B. monniera* standardized extract at dose 450 mg daily for 24 weeks showed significant improvement on the battery of neuropsychological tests, which indicated the positive effect on memory impairment in the elderly [21]. In addition, the *B. monniera* tablet showed improvement on working memory and attention after 12 weeks of treatment in healthy elderly volunteers. The possible mechanism of *B. monniera* was the suppression of plasma AChE activity [22]. The previous study of *B. monniera* standardized extract at dose 300 mg twice a day on Alzheimer's disease patients has reported that this drug could significantly increase MMSE score from 19.15 ± 4.54 to 23.85 ± 7.61 after six months of treatment [23].

Previous studies demonstrated that cholinesterase inhibitors had not shown a benefit to mild cognitive impairment. This current result was consistent with the large trial of donepezil for mild cognitive impairment treatment in which the gap of MMSE score between two groups narrowed and no significant differences could be identified [24]. Moreover, the InDDEx study has assessed the effect of rivastigmine, which is a cholinesterase inhibitor, on mild cognitive impairment patients. There was no significant difference in the cognitive test battery between the rivastigmine and placebo groups [25]. Galantamine did not show a significantly positive effect on mild cognitive impairment patients in terms of conversion to dementia [26].

There are several possible reasons for the lack of efficacy of KBD when compared to placebo in this study. This overall nonsignificant result of the KBD efficacy might be effected by the high education level of participants. The findings may not have a significance outcome because of the lack of a significant cholinergic deficit in this younger MCI population. Over a short period, the use of the mild cognitive impairment population might not offer sufficient severity to present a solid response to active treatment. In addition, this result could be the temporal effect of placebo treatment in subjects with cognitive impairment [27].

No patients treated with KBD dropped out of trials because of adverse effects. Treatment with KBD did not produce any severe side effects. The adverse events reported in both KBD and placebo subjects include burning sensations in the throat, dizziness, and drowsiness, none of which are considered harmful. The burning sensations in the throat could be effected by *P. nigrum*. A previous study showed certainly burning sensation effect like capsaicin via an agonist at the human TRPV1 receptor [28]. Drowsiness may occur from *C. asiatica* component, which is reported in the previous study as probably related to oral administration in humans [29]. Thus, the KBD formula modification should be developed to reduce the possible side effect. Our study found no changes in the blood chemistry of liver and kidney function. These findings were consistent with the prior study. In the previous study, the *in vivo* test of *N. nucifera* showed no acute toxicity in mice at lower 5 g/kg [30]. A clinical trial of *N. nucifera* in type 2 diabetes mellitus has been reported to effectively lower blood sugar without any unwanted hypoglycemia events [31]. The results of the preclinical safety assessment of *C. asiatica* extract were found safe in acute oral toxicity, subchronic toxicity, and mutagenicity in rats [32]. The clinical trial of *C. asiatica* oral dose was suggested at 250 mg and 500 mg to be safe in healthy volunteers [29]. *P. nigrum* was found to be safe in rats by showing nontoxicity in the acute phase (14 days) and subchronic phase (90 days) [33]. Even piperine from *P. nigrum* extract has been reported for increased AST level causing liver damage in albino mice; the present findings suggest that *P. nigrum* component in KBD cannot alter the liver enzyme in the trial period. These current results showed that the short-term safety of KBD in dose is about 2 g/day. A longer-term study (longer than six months) is needed to extend the safety results.

However, the current study has many limitations. First, the follow-up duration was short (3 months) and the sample size was small (*n* = 40). Usually, the duration of the clinical trial in mild cognitive impairment patients ranges from 6 to 18 months. In the present study, this is due to an ethical reason for patients who will be randomized to the placebo group, so this study is processed in a short duration compared to the disease progression process. This is the first trial of KBD in patients with mild cognitive impairment diagnosed according to MoCA score. However, our findings may not apply to some patients with mild cognitive impairment diagnosed by other criteria. The possession of the APOE *ε*4 allele should be performed in the study to predict the risk of progression to AD and to confirm the enrollment criteria of mild cognitive impairment.

Additionally, the amyloid positron emission tomography should be performed to assess the progression of the disease. We suggested studying in the elderly population for a possible significant effect on cognitive improvement. Although our findings do not support a clear recommendation for the use of KBD in mild cognitive impairment patients, a larger trial is urgently needed to explore the magnitude of efficacy.

## 5. Conclusions

To our knowledge, this is the first study to demonstrate evidence-based medicine with many biological parameters to support the efficacy and safety of the Kleeb Bua Daeng formula in mild cognitive impairment patients. Although we found no difference in overall benefit on the cognitive improvement of KBD compared with placebo, the safety profile of this trial suggests that KBD can be used safely in mild cognitive impairment patients with the underlying disease throughout the three months of the study. Unfortunately, this current study was underpowered to conclude the efficacy. A larger trial with longer duration needs to be performed. Lastly, the precise mechanisms of KBD to these effects still require further exploration.

## Figures and Tables

**Table 1:** Baseline characteristics of the KBD clinical trial in general.

	KBD	Placebo	*p* value
Male (%)	5 (25)	4 (20)	1.000
Female (%)	15 (75)	16 (80)	
Mean age ± SD (years)	58.30 ± 5.68	58.35 ± 6.65	0.980
Min	47	41	
Max	70	70	
Baseline blood pressure (mmHg)			
Systolic (mean ± SD)	129.25 ± 19.94	132.45 ± 17.30	0.5909
Min	103	100	
Max	163	174	
Diastolic (mean ± SD)	77.95 ± 11.01	75.40 ± 14.35	0.5322
Min	60	48	
Max	99	106	
BMI (mean ± SD)	24.06 ± 2.85	24.92 ± 5.23	0.5212
Min	17.76	16.06	
Max	29.97	32.98	

**Table 2:** Baseline characteristics of the KBD clinical trial for education, underlying diseases, average pill count, and MoCA mean score.

	KBD	Placebo	*p* value
Education (mean ± SD) (years)	11.65 ± 5.20	10.35 ± 5.06	0.4281
Min	4	4	
Max	18	18	
Underlying diseases			
Noninsulin-dependent	2	1	
Disorder of lipoprotein	3	2	
Heart disease	1	0	
Hypertension	4	2	
Gonarthrosis	0	1	
Functional dyspepsia	1	1	
Panic disorder	0	1	
Hyperplasia of prostate	0	1	
Average pill count capsules/month (mean ± SD)	111.7 ± 8.81	114.27 ± 7.82	0.100
MoCA score (mean ± SD)	21.55 ± 1.70	21.75 ± 1.59	0.703
Min	19	19	
Max	24	24	

**Table 3:** The change of MoCA score between KBD and placebo.

MoCA score	Baseline	Month 1	Month 2	Month 3
KBD	21.55	23.80	25.22	25.78^*∗∗∗*^
SD	1.70	2.95	1.66	1.66
Min	19	18	23	23
Max	24	29	28	29
Placebo	21.75	23.60	24.55	25.55^*∗∗∗*^
SD	1.59	3.03	3.68	2.98
Min	19	18	15	18
Max	24	28	29	29

^*∗∗∗*^ *p* < 0.001 showed difference between MoCA score at baseline and month 3 within groups.

**Table 4:** Estimated effect of the difference between KBD and placebo at each visit.

Study month	Mean difference of MoCA score (95% confidence interval)	*p* value
Month 1	−0.20 (−1.92, 1.52)	0.820
Month 2	−0.652 (−2.40, 1.10)	0.466
Month 3	−0.207 (−1.96, 1.54)	0.817

The multilevel mixed-effect linear regression model was used in analyzing mean difference (95% CI).

**Table 5:** Mean difference level of serum AST and ALT between KBD and placebo groups.

Study month	Mean difference of AST (95% confidence interval)	*p* value	Mean difference of ALT (95% confidence interval)	*p* value
Month 1	0.75 (−2.66, 4.16)	0.666	−1.70 (−7.55, 4.15)	0.569
Month 2	1.15 (−2.33, 4.63)	0.517	−1.79 (−7.77, 4.18)	0.556
Month 3	0.03 (−3.48, 3.54)	0.985	−4.81 (−10.84, 1.21)	0.117

The multilevel mixed-effect linear regression model was used in analyzing mean difference.

**Table 6:** Mean difference level of serum creatinine between KBD and placebo groups.

Study month	Mean difference of serum creatinine (95% confidence interval)	*p* value
Month 1	−0.04 (−0.17, 0.09)	0.522
Month 2	0 (−0.13, 0.13)	0.996
Month 3	−0.041 (−0.17, 0.09)	0.530

The multilevel mixed-effect linear regression model was used in analyzing mean difference.

**Table 7:** Mean difference level of eGFR between KBD and placebo groups.

Study month	Mean difference of eGFR (95% confidence interval)	*p* value
Month 1	2.40 (−7.52, 12.33)	0.635
Month 2	0.123 (−9.89, 10.13)	0.981
Month 3	1.844 (−8.17, 11.85)	0.718

The multilevel mixed-effect linear regression model was used in analyzing mean difference.

**Table 8:** Adverse events of KBD.

Adverse effect	KBD	Placebo
Burning sensation in the throat (%)	3 (16.7)	7 (35)
Dizziness (%)	1 (5.6)	0
Drowsiness (%)	1 (5.6)	0
Increased appetite (%)	1 (5.6)	0

## Data Availability

The main data are included within the content. Other data are available from the corresponding author upon request.
